# Colistin Resistance in Klebsiella pneumoniae: Mechanisms, Detection, and Clinical Impact

**DOI:** 10.7759/cureus.112074

**Published:** 2026-07-05

**Authors:** Sai J Koli, Geeta S Karande, Vinayak S Bhosale, Satish R Patil

**Affiliations:** 1 Department of Microbiology, Krishna Institute of Medical Sciences, Krishna Vishwa Vidyapeeth (Deemed to be University), Karad, IND; 2 Krishna Charitable Trust, Krishna Institute of Medical Sciences, Krishna Vishwa Vidyapeeth (Deemed to be University), Karad, IND

**Keywords:** antimicrobial resistance, carbapenem resistance, colistin resistance, klebsiella pneumoniae, mcr-1, molecular characterization

## Abstract

*Klebsiella pneumoniae* (*K. pneumoniae* ​​​​​) is a Gram-negative, encapsulated opportunistic pathogen causing pneumonia, urinary tract infections, bloodstream infections, and ventilator-associated pneumonia. Carbapenem resistance has made colistin a last-resort antibiotic for multidrug-resistant Gram-negative infections, but increased colistin use has driven resistance emergence. Resistance occurs through chromosomal mutations (mgrB, phoPQ, pmrAB) causing lipid A modification that reduces colistin binding, and plasmid-mediated mcr genes (especially mcr-1) encoding phosphoethanolamine transferase that spread horizontally across strains and species, making it a critical public health concern. Broth microdilution is the reference standard for susceptibility testing, while disk diffusion is unreliable. Molecular methods (polymerase chain reaction (PCR), sequencing) detect resistance genes and distinguish chromosomal versus plasmid-mediated mechanisms, providing comprehensive characterization for surveillance and outbreak investigation.

Colistin-resistant *K. pneumoniae* is predominantly healthcare-associated, linked to ICU admission, prolonged hospitalization, broad-spectrum antibiotic exposure (carbapenems, colistin), and invasive devices (central venous catheters, urinary catheters, ventilators). Infections cause delayed effective therapy, prolonged hospital stay, increased complications, and higher mortality, particularly in critically ill patients with bloodstream infections or sepsis. India faces a high burden of carbapenem- and colistin-resistant isolates in tertiary care hospitals, yet molecular characterization data from regional hospitals remain limited. Rapid molecular diagnostics detecting mcr genes enable earlier appropriate therapy. Antimicrobial stewardship combined with infection control measures (contact precautions, hand hygiene, active surveillance, environmental cleaning) is essential to preserve colistin effectiveness and limit resistance spread, maintaining it as a last-line therapeutic option.

## Introduction and background

*Klebsiella pneumoniae *(*K. pneumoniae*) is a Gram-negative, encapsulated, opportunistic pathogen that causes a wide range of infections in both community and hospital settings. It is an important cause of pneumonia, urinary tract infections, bloodstream infections, wound infections, and ventilator-associated pneumonia. Recently, the organism has become a major concern due to its ability to acquire multiple antimicrobial resistance mechanisms and to survive in hospital environments [1].

Antimicrobial resistance in *K. pneumoniae *has emerged as a serious global problem. The organism now shows resistance to multiple frequently used antibiotic classes, including cephalosporins, fluoroquinolones, aminoglycosides, and carbapenems. The rise of carbapenem-resistant *K. pneumoniae* has significantly reduced therapeutic options and has made treatment of severe infections more difficult. As a result, older reserve drugs such as colistin have regained importance in the management of multidrug-resistant Gram-negative infections [2].

Colistin is considered a last‑resort antibiotic for carbapenem‑resistant Enterobacterales (CRE) infections. It binds to the outer membrane of Gram‑negative bacteria and disrupts membrane integrity. However, increased and often inappropriate use of colistin has led to the emergence of colistin resistance. This resistance is clinically significant because it compromises one of the few remaining treatment options for highly resistant bacterial infections [3].

Colistin resistance in *K. pneumoniae* (CRKP) occurs through both chromosomal and plasmid-mediated mechanisms. Chromosomal resistance commonly involves alterations in regulatory systems such as mgrB, phoPQ, and pmrAB, which lead to modifications of lipid A and reduced colistin binding. Plasmid-mediated resistance is mainly associated with mcr genes, which can spread horizontally between bacteria and contribute to rapid dissemination of resistance. The coexistence of these mechanisms makes the organism more difficult to control in hospital settings [4].

Detection of colistin resistance is an important part of laboratory diagnosis. Broth microdilution is considered the reference method for colistin susceptibility testing and determination of the minimum inhibitory concentration (MIC), as many routine methods may give unreliable results. Molecular methods such as polymerase chain reaction (PCR) are useful for identifying resistance genes and understanding the genetic basis of resistance. Combining phenotypic and genotypic approaches provides a clearer picture of resistance patterns and helps in accurate surveillance [5].

The spread of colistin-resistant *K. pneumoniae* is strongly associated with hospital-related risk factors such as intensive care unit (ICU) admission, prolonged hospitalization, prior exposure to broad-spectrum antibiotics, and use of invasive devices. These factors create selective pressure and facilitate transmission of resistant strains among vulnerable patients. As a result, infection control and antimicrobial stewardship play a critical role in limiting spread [6].

In India, colistin resistance has become an increasing concern because of the high burden of multidrug-resistant infections, frequent antibiotic use, and limited treatment alternatives in many tertiary care hospitals. Although several studies have reported colistin resistance from different parts of the country, detailed molecular data from many regional hospitals are still limited. This creates an important knowledge gap regarding the local distribution of resistance mechanisms and the epidemiology of resistant strains [7].

Therefore, the study of colistin-resistant *K. pneumoniae *is important for understanding the burden, mechanisms, risk factors, and clinical significance of resistance in hospital settings. Such information can support improved diagnostic strategies, guide rational antibiotic use, strengthen infection control measures, and contribute to antimicrobial stewardship programs [8].

Methodology

Search Strategy

A comprehensive literature search was conducted in accordance with the Preferred Reporting Items for Systematic Reviews and Meta-Analyses (PRISMA) 2020 guidelines [9]. Electronic databases including PubMed, Scopus, Web of Science, ScienceDirect, and Google Scholar were systematically searched for relevant studies published between 2011 and 2026. Additional articles were identified through manual screening of the reference lists of eligible studies.

The search strategy included combinations of the following keywords and Medical Subject Headings (MeSH) terms: "*Klebsiella pneumoniae,*" "colistin resistance," "polymyxin resistance," "mcr gene," "mcr-1," "carbapenem-resistant *Klebsiella pneumoniae,*" "multidrug-resistant *Klebsiella pneumoniae,*" and "antimicrobial resistance." Boolean operators (AND, OR) were used to optimize the search strategy.

After removal of duplicate records, titles and abstracts were screened for relevance, and full-text articles were assessed based on predefined inclusion and exclusion criteria. Relevant data, including study design, sample size, prevalence of colistin resistance, resistance mechanisms, and key findings, were extracted from eligible studies. The quality of included studies was evaluated using appropriate risk-of-bias assessment criteria according to the study design.

Inclusion criteria comprised original research articles, observational studies, molecular epidemiological studies, surveillance studies, systematic reviews, and review articles published in English that reported data on CRKP, including resistance mechanisms, laboratory detection methods, epidemiology, risk factors, clinical outcomes, infection control measures, and antimicrobial stewardship. These diverse sources were included to provide a comprehensive overview of the epidemiology, mechanisms, detection methods, and clinical impact of colistin-resistant *K. pneumoniae*.

Exclusion criteria included conference abstracts without full text, case reports, editorials, letters to the editor, non-English publications, duplicate publications, studies lacking sufficient microbiological or molecular data, and articles not specifically related to colistin-resistant *K. pneumoniae*. 

**Figure 1:**
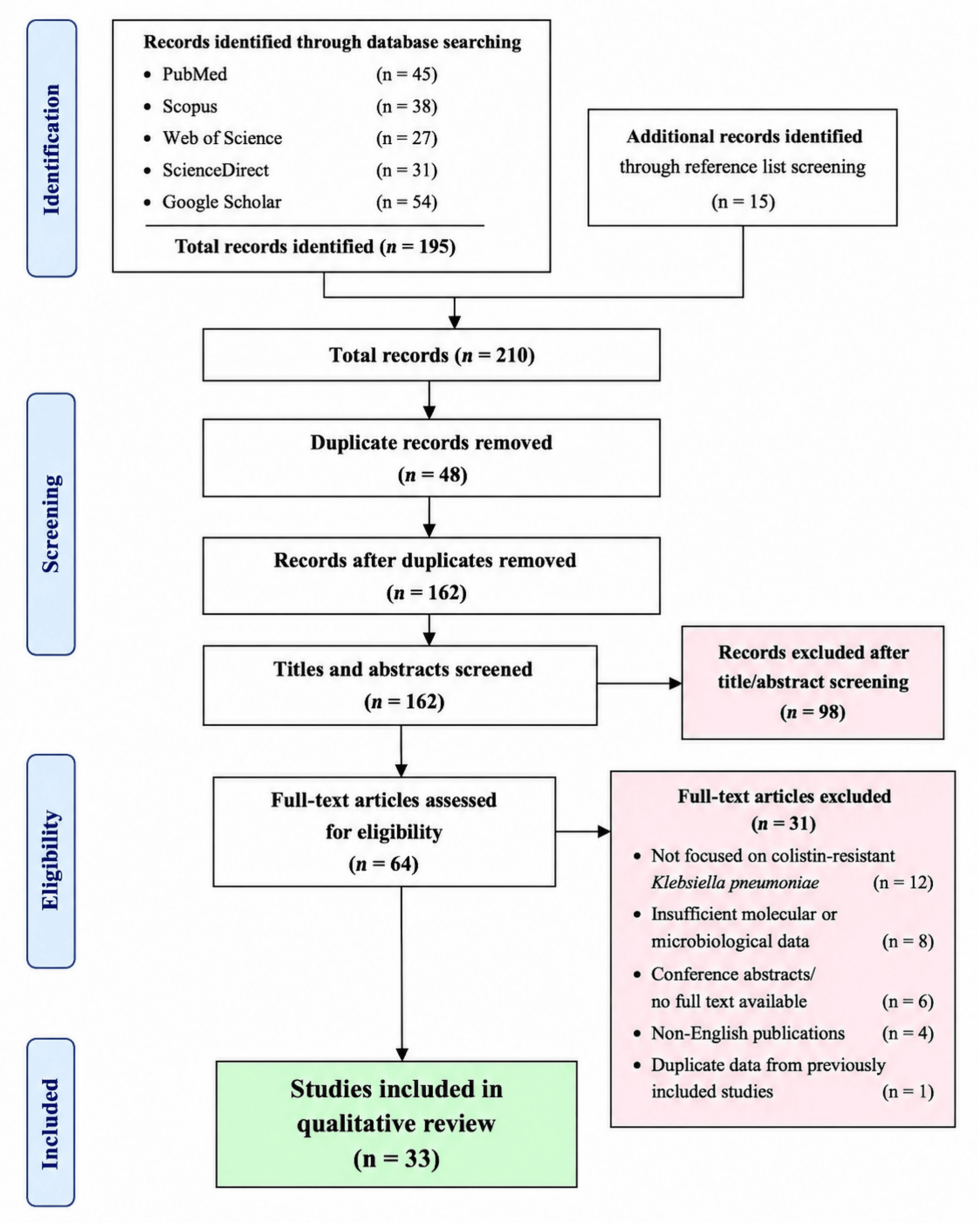
PRISMA flow diagram n: number; PRISMA: Preferred Reporting Items for Systematic Reviews and Meta-analyses

A total of 210 records were identified through database searching (195 records) and reference list screening (15 records). After removing 48 duplicate records, 162 records remained for title and abstract screening. Of these, 98 records were excluded. The full texts of 64 articles were assessed for eligibility, and 31 articles were excluded due to reasons such as lack of focus on *K. pneumoniae*, insufficient microbiological data, conference abstracts, non-English publications, and duplicate data. Finally, 33 studies were included in the qualitative review (Figure 1) [9].

## Review

Colistin as a reserve drug

Colistin is a polymyxin antibiotic considered a last‑resort or reserve drug for severe infections due to multidrug‑resistant Gram‑negative bacteria. It is particularly important in infections due to carbapenem-resistant *K. pneumoniae*, *Acinetobacter baumannii, *and *Pseudomonas aeruginosa *when other antimicrobial agents have become ineffective. Colistin acts by binding to the lipopolysaccharide layer of the outer membrane of Gram-negative bacteria, disrupting membrane integrity, increasing permeability, and causing leakage of intracellular contents, which ultimately leads to bacterial death. Despite this unique mechanism, its clinical use is limited by notable adverse effects, particularly nephrotoxicity, which require careful dosing and close monitoring, especially among critically ill patients and those with renal impairment [10].

In addition, colistin has a narrow therapeutic window, making it difficult to balance efficacy and toxicity. Because of these limitations, dose adjustment and, where available, monitoring drug levels are important during treatment to optimize dosing. Colistin also has historical significance because it re-emerged into clinical use after the rise of antimicrobial resistance reduced the effectiveness of many newer antibiotics. In severe infections, it is sometimes used as part of combination therapy to improve outcomes and reduce the chance of treatment failure. Although renewed interest in colistin has emerged because of the growing burden of antimicrobial resistance, its usefulness is increasingly threatened by the emergence of resistance, mediated by chromosomal mutations and plasmid-borne mcr genes. Therefore, colistin should be used judiciously, only when clearly indicated, and as part of antimicrobial stewardship, laboratory surveillance, and infection control measures to preserve its effectiveness [11].

Resistance mechanisms: chromosomal and plasmid-mediated

Colistin resistance in *K. pneumoniae* develops mainly through chromosomal alterations and plasmid-mediated mechanisms. Chromosomal resistance commonly involves mutations or inactivation of regulatory systems such as mgrB, phoPQ, pmrAB,and occasionally crrAB*.* These genetic changes alter the bacterial outer membrane by modifying lipid A, usually through the addition of phosphoethanolamine or 4-amino-4-deoxy-L-arabinose, which reduces the negative charge of the cell surface and weakens the binding of colistin. As a result, the antibiotic cannot interact effectively with the outer membrane and loses its bactericidal activity [12].

Plasmid-mediated resistance is most often associated with the mcr gene family, especially mcr-1, although additional variants have been identified. These genes code for phosphoethanolamine transferase enzymes that produce a similar lipid A modification, leading to decreased colistin susceptibility. This form of resistance is particularly concerning because plasmids can move rapidly between bacteria by horizontal gene transfer, allowing resistance to spread across strains, species, and hospital environments. This makes plasmid-mediated resistance an important epidemiological and public health problem [13].

In addition to these major mechanisms, colistin resistance may also be influenced by the coexistence of multiple resistance determinants within the same isolate. Some strains show a combination of chromosomal mutations and plasmid-mediated genes, which may result in higher levels of resistance and more complex phenotypes. Such isolates are especially difficult to treat and may also show association with other antimicrobial resistance determinants, further limiting therapeutic options. Therefore, detailed understanding of both chromosomal and plasmid-mediated pathways is essential for accurate laboratory detection, epidemiological surveillance, and effective control of colistin-resistant *K. pneumoniae* [14].

Laboratory detection methods

Laboratory detection of CRKP requires both phenotypic and genotypic approaches. Phenotypic testing determines whether the isolate is susceptible or resistant, while molecular methods help identify the underlying resistance mechanism. Because colistin testing is technically difficult and results can vary with the method used, selecting the correct test is essential for reliable reporting [15].

Broth microdilution is considered the reference standard for colistin susceptibility testing. It provides the MIC and is preferred over disk diffusion, which is unreliable for colistin. In routine practice, isolates are categorized as resistant, intermediate, or susceptible according to accepted interpretive breakpoints. This method is especially useful because it gives reproducible results and is suitable for confirmatory testing [16].

Other phenotypic methods may include routine screening for carbapenem resistance and confirmation of carbapenemase production by methods such as the modified carbapenem inactivation method (mCIM). Since colistin resistance is often seen in carbapenem-resistant isolates, initial phenotypic screening helps identify the strains that should be further evaluated. This stepwise strategy is practical in hospital microbiology laboratories and helps reduce unnecessary testing [16].

Genotypic detection is performed by PCR-based amplification of resistance genes such as mgrB and mcr variants. These tests help determine whether resistance is due to chromosomal changes or plasmid-mediated genes. In some studies, sequencing is also used to detect mutations in regulatory genes and to define the exact molecular basis of resistance. Molecular methods are particularly valuable for surveillance, outbreak investigation, and phenotype-genotype correlation [17].

In addition, molecular testing can identify resistance even when phenotypic results are unclear or borderline. This is useful in research settings and in hospital surveillance programs where detailed characterization is required. Therefore, combining phenotypic and genotypic methods provides the most complete understanding of colistin resistance in clinical isolates [18].

Risk factors and hospital epidemiology

CRKP* *is predominantly a healthcare-associated pathogen and is commonly recovered from critically ill patients and those with prolonged hospitalization. Important risk factors include ICU admission, prolonged hospitalization, prior exposure to broad-spectrum antibiotics such as carbapenems or colistin, and the use of invasive devices including central venous catheters, urinary catheters, ventilators, and endotracheal tubes. These conditions exert strong selection pressure, leading to the emergence and persistence of resistant organisms [19].

Hospital epidemiology plays a key role in understanding how this pathogen spreads within healthcare facilities. Transmission may occur through direct patient contact, contaminated hands of healthcare workers, shared instruments, or environmental surfaces that become colonized. Once introduced into a ward, especially in high-risk areas such as ICUs, surgical units, or oncology wards, resistant strains may spread rapidly and cause clusters or outbreaks. The presence of mobile resistance elements further increases the potential for dissemination [20].

Patients with severe illness, immunosuppression, mechanical ventilation, or indwelling lines are particularly susceptible to colonization and infection. These patients often have repeated antibiotic exposure and impaired host defenses, which increase the risk of acquiring resistant strains and also make treatment more difficult. In addition, colonization may precede infection, allowing resistant organisms to persist silently within the hospital environment. Therefore, understanding the epidemiology and risk profile of colistin-resistant *K. pneumoniae* is essential for prevention, early detection, and infection control [21].

Clinical outcomes and infection control

Colistin-resistant *K. pneumoniae* is associated with unfavorable clinical outcomes because treatment options become severely limited once resistance develops. Infections caused by these strains are frequently associated with delayed effective therapy, prolonged hospital stay, more complications, and higher mortality, particularly in critically ill patients and those with bloodstream infections. The situation becomes more serious when the isolate is resistant to multiple antibiotic classes, leaving very few therapeutic choices [22].

Evidence from several studies suggests that central venous catheter use, previous antibiotic exposure, ICU admission, and higher severity scores are associated with worse outcomes in patients infected with colistin-resistant strains. The risk is also higher in patients with sepsis, ventilator-associated pneumonia, or underlying comorbidities such as diabetes, renal disease, or immunosuppression. Once resistance appears, even reserve drugs may fail, making management much more difficult and increasing the risk of clinical deterioration. In addition, treatment delays may allow the infection to progress before appropriate therapy is started [21].

Infection control is essential to prevent spread within hospitals. Important measures include contact precautions, strict hand hygiene, active surveillance of high-risk patients, cleaning of the environment, and prompt isolation of colonized or infected cases. Screening of contacts and early identification of carriers can also help prevent silent transmission within wards. Antimicrobial stewardship is equally crucial because reducing unnecessary antibiotic exposure helps limit further selection of resistant organisms [23].

A coordinated infection control plan should also include staff education, proper disinfection of shared equipment, and regular surveillance of resistance trends in the hospital. Communication between the microbiology laboratory, clinicians, and infection control team is important for timely intervention. These combined measures help reduce transmission, improve patient safety, and preserve the effectiveness of remaining antibiotics [24].

Indian scenario and knowledge gap

In India, colistin-resistant *K. pneumoniae* is an increasing public health concern because tertiary care hospitals carry a heavy burden of carbapenem-resistant Enterobacterales and often use broad-spectrum antibiotics extensively. Reports from several Indian centers show that carbapenem-resistant and colistin-resistant isolates are frequently recovered from ICUs, surgical wards, and other high-risk units, suggesting strong hospital-based transmission and selection pressure. The problem is particularly important in patients who have prolonged hospital stays, repeated antibiotic exposure, and severe underlying illness [25].

The Indian scenario is more complex because resistance patterns differ significantly across regions, hospitals, and patient populations. Some centers report high rates of colistin resistance among carbapenem-resistant isolates, while others show lower but steadily rising levels. This variation reflects differences in antibiotic use, infection control practices, patient referral patterns, and local microbial ecology. As a result, national data alone may not reflect the resistance burden in individual hospitals [26].

A major knowledge gap is the limited molecular characterization of resistant isolates from many regional and smaller tertiary care hospitals. Most available studies describe phenotypic resistance, but fewer investigations explore the exact genetic basis, such as mgrB inactivation, mcr genes, or other regulatory mutations. Information about clonal relatedness, plasmid dissemination, and co-existence of virulence and resistance factors is also sparse. Without this molecular detail, it is difficult to understand how resistance emerges and spreads in local settings. Another important gap is the lack of hospital-specific surveillance data that links laboratory findings with patient-level risk factors and outcomes. Such data are needed to identify local transmission patterns, high-risk wards, and effective intervention strategies. Therefore, more regional studies from India are essential to strengthen antimicrobial stewardship, improve infection control, and guide treatment decisions in a setting where colistin remains a critical reserve antibiotic [27].

Future directions: rapid diagnostics and stewardship

Future management of colistin-resistant *K. pneumoniae* will depend on faster, more accurate, and more accessible diagnostic methods. Rapid molecular tests can detect important resistance determinants such as mcr genes and mutations in regulatory genes at an early stage, allowing clinicians to start appropriate therapy sooner. This is especially important in severe infections, where delay in effective treatment can worsen outcomes and increase mortality. In the future, point-of-care molecular platforms and automated resistance detection systems may further improve turnaround time and laboratory efficiency [28].

Although broth microdilution will remain the reference method for colistin susceptibility testing, newer rapid phenotypic and genotypic approaches may complement routine laboratory workflows. Faster detection in the microbiology laboratory can support earlier infection control measures, especially in ICUs, transplant units, and other high-risk wards. It can also help clarify borderline or uncertain results that may occur with some conventional methods. Integration of rapid diagnostics with hospital information systems may improve communication between the laboratory and clinicians [29].

Antimicrobial stewardship is equally important in limiting the spread of resistance. Rational use of colistin and other reserve antibiotics can reduce selective pressure and slow the emergence of resistant strains. Stewardship programs should encourage appropriate antibiotic selection, dose optimization, timely de-escalation, and regular review of culture and susceptibility results. In addition, education of clinicians about reserve antibiotic use can improve prescribing behavior and reduce unnecessary exposure [30].

Infection prevention measures must work together with diagnostics and stewardship to control transmission within hospitals. Surveillance, contact precautions, hand hygiene, environmental cleaning, and isolation of colonized or infected patients remain essential. Active monitoring of resistance trends and outbreak detection can help institutions respond quickly to emerging threats. Together, these strategies can improve patient outcomes and help preserve colistin as a last-line therapeutic option [23].

Virulence Factors of Colistin-Resistant K. pneumoniae

Colistin-resistant *K. pneumoniae* remains a significant clinical concern because certain resistant strains retain important virulence characteristics, including capsule production, biofilm formation, and efficient iron-acquisition systems. These factors enhance bacterial survival, persistence, and transmission in healthcare settings. Biofilm formation is particularly important because it promotes colonization of medical devices and may contribute to reduced antimicrobial susceptibility [31].

The coexistence of virulence and colistin resistance increases the clinical impact of *K. pneumoniae* infections. Hypervirulent and multidrug-resistant strains have been associated with severe infections, including bloodstream infections and pneumonia, while also limiting therapeutic options. Understanding the relationship between virulence determinants and colistin resistance is important for improving infection control, surveillance, and treatment strategies [32].

Treatment challenges

Treatment of colistin-resistant *K. pneumoniae* should be individualized according to the site of infection, antimicrobial susceptibility results, carbapenemase type, severity of illness, and local availability of active antimicrobial agents. These factors play a critical role in selecting the most appropriate therapeutic strategy and improving clinical outcomes. Treatment of colistin-resistant *K. pneumoniae* is difficult because the organism often shows resistance to multiple antibiotic classes, leaving very few effective options. When colistin resistance develops, even reserve therapy becomes unreliable, and clinicians may face delayed appropriate treatment, which can worsen patient outcomes. This becomes especially problematic in severe infections such as sepsis, ventilator-associated pneumonia, and bloodstream infection, where early effective therapy is critical for survival [2].

One major challenge is the lack of safe and highly effective alternatives for infections caused by multidrug-resistant or carbapenem-resistant strains. In many cases, treatment may require combination therapy, careful dose adjustment, and close monitoring of the patient. Toxicity of available drugs, especially nephrotoxicity with colistin, further complicates management. In addition, pharmacokinetic variability in critically ill patients can make it difficult to achieve effective drug concentrations without increasing adverse effects [3].

Another problem is that resistance mechanisms can vary between isolates. Some strains carry chromosomal mutations, while others harbor plasmid-mediated resistance genes, and some may have both. This genetic diversity makes treatment prediction difficult and highlights the need for laboratory confirmation before therapy. It also raises concern for treatment failure if therapy is started without knowing the exact susceptibility pattern [33].

Because of these challenges, antimicrobial stewardship and infection control are very important. Rational antibiotic use, early detection of resistant isolates, and prevention of hospital spread can help preserve the remaining treatment options and improve outcomes. In addition, clinicians often need to balance the urgency of treatment with the limited evidence available for some salvage regimens, which makes expert consultation useful in complex cases.

## Conclusions

Colistin-resistant *K. pneumoniae* is an emerging public health concern due to limited treatment options and increasing antimicrobial resistance. Resistance is primarily mediated by chromosomal mutations and plasmid-borne mcr genes, while healthcare-associated factors contribute to its spread. Accurate detection, antimicrobial stewardship, and strict infection-control measures are essential for effective management. Continued surveillance and molecular characterization, particularly in India, are needed to understand resistance patterns and support strategies to prevent further dissemination of resistant strains.
